# *Ocimum sanctum*, *Zingiber officinale,* and *Piper nigrum* extracts and their effects on gut microbiota modulations (prebiotic potential), basal inflammatory markers and lipid levels: oral supplementation study in healthy rats

**DOI:** 10.1080/13880209.2022.2033797

**Published:** 2022-02-19

**Authors:** Narendra Babu Kondapalli, Rajkumar Hemalatha, Satyanarayana Uppala, Srinivas Reddy Yathapu, Shujauddin Mohammed, Mullapudi Venkata Surekha, Ananthan Rajendran, Dinesh Kumar Bharadwaj

**Affiliations:** a Department of Microbiology & Immunology, ICMR-National Institute of Nutrition, Hyderabad, Telangana State, India; b Dr. Pinnamaneni, Siddhartha Institute of Medical Sciences, Vijayawada, Andhra Pradesh, India; c Department of Pathology, ICMR-National Institute of Nutrition, Hyderabad, Telangana State, India; d Food and Drug Toxicology department, ICMR-National Institute of Nutrition, Hyderabad, Telangana State, India; e Food Chemistry Division, ICMR-National Institute of Nutrition, Hyderabad, Telangana State, India

**Keywords:** Herbal extracts on endotoxin, beneficial bacteria, systemic inflammation, phenolic compounds, essential oils

## Abstract

**Context:**

*Ocimum sanctum* Linn (Labiatae) (OS), *Zingiber officinale* Rose (Zingiberaceae) (ZO), and *Piper nigrum* Linn (Piperaceae) (PN) are used in traditional medicine as immunomodulator, anti-inflammatory, and bioavailability enhancer agents.

**Objective:**

Active phytoconstituents of OS, ZO, PN hydro-alcoholic extracts and their effects on gut microbiota, basal inflammation and lipid profile were investigated in rats.

**Materials and methods:**

Active phytoconstituents of extracts were analysed using HPLC and GC-MS. SD rats were supplemented with individual/combined extracts (OS-850; ZO-500; PN-100 mg/kg Bw) and Fructooligosaccharide (standard prebiotic-5g/kg-Bw), orally for 30 days. Haematology, lipid profile, LPS, CRP, IL-6, insulin and histology of vital organs were analysed. Caecal bacterial levels were assessed by RT-PCR.

**Results:**

High content of phenolic compounds luteolin-7-*O*-glucoside (430 ± 2.3 mg/100g), gallic acid (84.13 ± 1.2 mg/100 g) and flavones (88.18 ± 1.8 mg/100 g) were found in OS, ZO, and PN, respectively. Combined extract was rich in luteolin-7-*O*-glucoside (266.0 ± 1.80 mg/100 g). Essential oils including methyleugenol (13.96%), 6-shogaol (11.00%), piperine (18.26%), and cyclopentasiloxane (10.06%) were higher in OS, ZO, PN and combined extract. Higher levels of caecal *Lactobacillus* (1.7–3.4-fold), *Bifidobacterium* (5.89-28.4-fold), and lower levels of *Firmicutes* (0.04–0.91-fold), *Bacteroides* (0.69–0.88-fold) were noted among extracts and FOS supplemented rats. Significant (*p <* 0.05) decrease in plasma lipid profile and LPS was noted in all supplemented rats.

**Discussion and conclusions:**

The current study could be first of its kind in exploring prebiotic potential of OS, ZO, PN and their effect on native gut bacterial population.

## Introduction

Recently, traditional medicine has gained attention with the scientific validation of the proposed properties of medicinal/spice/herbal based products. The use of medicinal herbs is well documented in Ayurveda, Siddha, and Chinese systems of medicine. In addition, Unani, Greek, and Roman systems of medicine are attaining popularity for many diseases including common cold and a variety of non-communicable diseases. The therapeutic potential of *Ocimum sanctum* Linn (Labiatae) is documented in Ayurveda and Siddha for its healing properties. Whereas Greek, Roman and Unani systems of medicines indicated its role in treatment of skin diseases, common cold, headache, cough, and malaria. Therapeutic properties of *O. sanctum, Zingiber officinale* Rose (Zingiberaceae), and *Piper nigrum* Linn (Piperaceae) are widely reported and major components of these three herbs are well characterized and are also used in immune and inflammatory conditions such as diabetes, obesity, asthma (Damanhouri and Ahmad 2014; de Lima et al. 2018; Singh and Chaudhuri 2018). The plethora of scientific literature and classical Ayurvedic text suggests that the herbs *O. sanctum*, *Z. officinale,* and *P. nigrum* have a wide variety of proven functions and specific properties such as immunomodulatory, anti-inflammatory, and ability to increase the bioavailability of active compounds, respectively (Alizadeh-Navaei et al. 2008; Damanhouri and Ahmad 2014; Parasuraman et al. 2015). It is noteworthy to mention that these herbal extracts possess a vast number of ‘phytochemical constituents’ whose bioactivities were assigned to such active principal ingredients (APIs).

Chronic low grade systemic inflammation, following translocation of endotoxin (lipopolysaccharide), is the underlying pathogenesis of chronic non-communicable diseases (NCD) (Cani et al. 2008). It is well known fact that the basal endotoxin [i.e., lipopolysaccharide (LPS)] levels of healthy humans are influenced by a wide range of factors such as dietary composition, stress, low grade inflammation, altered gut flora, etc. (Lyte et al. 2016). The critical role of gut bacteria has been suggested through intestinal membrane integrity and control of translocation of endotoxin into circulation (Cani et al. 2008). The balance between beneficial bacteria such as *Lactobacillus*, *Bifidobacterium,* etc. and *Firmicutes*, *Bacteroides* are considered as an important aspect for basal endotoxemia (Cani et al. 2008).

Prebiotics are non-digestible fermentable fibres that promote health by modifying the intestinal microflora towards more protective intestinal bacteria (Markowiak and Śliżewska 2017). Multi-dimensional actions were attributed to prebiotics, which includes improvement of intestinal membrane integrity; regulation of systemic and mucosal immune responses of the host along with amelioration of inflammation (Markowiak and Śliżewska 2017). Recent studies conducted with extracts of *O. sanctum, Z. officinale,* and *P. nigrum* have shown a significant reduction in systemic inflammation (Tasleem et al. 2014; Hsiang et al. 2015; Singh and Chaudhuri 2018) whereas their prebiotic potential is not explored to date.

The *in vitro* studies from our laboratory have proven the growth promoting prebiotic potential of *O. sanctum, Z. officinale,* and *P. nigrum* extracts on gut beneficial bacteria viz *Lactobacillus* and *Bifidobacterium*, which has been attributed to their phytochemical constituents (Babu et al. 2018). In ancient days, the active principal ingredient responsible for such medicinal activity was not explored, however a wider acceptance of herbal based medicine demands the scientific proof of concept using advancement of technology *vis-à-vis* with phytoconstituents and their functions. Most of herbs/medicinal plants are rich in phytochemicals such as phenolics, flavonoids, terpenoids, tannins, antioxidants, fibre, etc. which have a pivotal role in combating diseases. These ingredients are known to modulate the normal flora, especially gut microbiota when these medicinal/herbal extracts are administered orally (Duenas et al. 2015). Researchers demonstrated the growth promoting effects of tea polyphenols, red wine, polyphenol powder, apple juice, resveratrol, blackcurrant extracts with abundant growth of *Lactobacillus, Bifidobacterium* and inhibitory action on *Bacteroides* (Duenas et al. 2015). An association has been established between improved beneficial gut microbiota and metabolic disease status through key pathways of energy homeostasis and inflammation (Cani et al. 2008). Further, our *in vitro* data pertaining to prebiotic potential of *O. sanctum, Z. officinale,* and *P. nigrum* extracts strongly suggested the proliferative effect on certain gut microbiota (Babu et al. 2018). But, none of the studies reported the role of these herbs for prebiotic potential so far, although prebiotics are known to improve insulin sensitivity and reduce inflammatory markers similar to these herbs (Musso et al. 2010).

In the recent past, the *in vivo* studies conducted with extracts of *O. sanctum, Z. officinale,* and *P. nigrum* reported no changes in hematological profile of healthy animals even upon prolonged administration (Rong et al. 2009; Sriwiriyajan et al. 2016; Singh and Chaudhuri 2018). However, *O. sanctum, Z. officinale,* and *P. nigrum* extracts were known to regulate the lipid metabolism, insulin resistance and inflammatory markers specially C-Reactive Protein (CRP), LPS and IL-6 levels (Alizadeh-Navaei et al. 2008; Damanhouri and Ahmad 2014; Parasuraman et al. 2015).

It is clear from the above background that the *in vivo* animal model and clinical studies exhibited immune-inflammatory modulation by the extracts of *O. sanctum, Z. officinale,* and *P. nigrum*. In view of this and due to the lack of data on prebiotic potential of these herbs on selective gut microbiota and its corresponding consequences in normal healthy individuals, the present study aimed to investigated the same in healthy Sprague-Dawley (SD) rats.

## Materials and methods

### Collection of plant materials

The plant materials selected for current study were dried leaves of *O. sanctum* (Heritage Bio-natural Systems Pvt. Ltd), dried rhizome of *Z. officinale,* and seeds of *P. nigrum* which were procured (October 2015) from an herbal store (Balmukand DevkaranSharada) at Hyderabad, India. The voucher specimens were submitted to the Department of Botany, Osmania University, Hyderabad, India and authenticated (Prof. Rana Kausar, Head, Department of Botany) certificates were obtained for the same.

### Preparation of extracts

The process and rationale for selection of hydro-alcoholic extracts (HA) of *O. sanctum, Z. officinale* and *P. nigrum* were as reported earlier from our lab (Babu et al. 2018). Further, the HA extracts of chosen herbs were authenticated by quantification of active ingredients using HPLC analysis along with reporting the preliminary phytochemical screening, and antioxidant activities (Babu et al. 2018).

### Analysis of phenolic compounds in hydro-alcoholic extracts

The phenolic compound contents in individual and combined hydro-alcoholic extracts were quantified on C18 column (100 × 2.1 mm) of 3 μm, using diode array detection (DAD) system (DIONEX). Different proportions of solvents such as 90% phosphate buffer (50 mM pH3.3) and 10% methanol as eluent A, 30% phosphate buffer (50 mM pH3.3) and 70% methanol as eluent B were used for separation. The mobile phase flow rate was 0.47 mL/min with run time of 52 min and column oven temperature was maintained at 35 °C. The chromatogram was monitored at 250, 280, 320, and 370 nm. The peak purity of the tested sample was determined by comparing its ultraviolet (UV) spectra to that of the reference standards.

### Analysis of essential oils in extracts by GC-MS method

The essentials oil percentage in hydro-alcoholic extracts of *O. sanctum*, *Z. officinale,* and *P. nigrum* alone and combined were analysed by GC-MS method (Agilent-Technologies model 5977). The essential oil chemical constituents of extracts were separated using a HP-5MS capillary column (30 m × 0.25 mm, film thickness 0.25 μm). A sample of 1.0 μL was injected (Split ratio 10:1) on helium as carrier gas at a flow rate of 1.2 mL/min. The initial column temperature was maintained at 50 °C for 2 min, with a programmed increment of 10 °C/min to 280 °C followed by hold at the same temperature for 5 min. The injection port, transfer line and source temperatures were 250 °C, 280 °C, and 230 °C, respectively. For GC-MS detection, an electron impact ionisation mode, with ionisation energy of 70 eV, was used. The different compounds present in the extracts were identified using the National Institute of Standards and Technology (NIST14) mass spectral library.

## Experimental design

### Rationale for selection of dosage

The dosages were selected based on our previous studies and existing literature pertaining to therapeutic potential of chosen herbs. A dose of 850 mg/kg-Bw of *O. sanctum* was selected, based on a series of our scientific studies, which established the potential activity in terms of improved antibody titre, Hb levels, etc. in healthy mice (Hemalatha et al. 2011). Similarly, the dose for *Z. officinale* (500 mg/kg-Bw) and *P. nigrum* (100 mg/kg-Bw) were selected keeping in view of widely reported therapeutic actions (Nirwane and Bapat 2012; Balogun et al. 2019). It is well established that each of the selected herbs have specific properties such as immunomodulatory, anti-inflammatory and improving the bioavailability of active compounds, thus the combination of three (i.e., *O. sanctum* 850 mg/kg-Bw *+ Z. officinale* 500 mg/kg-Bw and *P. nigrum* 100 mg/kg-Bw) extracts were also tested, to understand their additive effect, if any.

### Animals and treatment

Institutional and national guidelines for the care and use of animals were followed and all experimental procedures involving animals were approved (No: P18 F/IAEC/NIN/2013/RH/SD-F-56) by the IAEC (Institutional Animal Ethical Committee) of the ICMR- National Institute of Nutrition, Hyderabad, India.

Three months old, female Sprague-Dawley rats (*n* = 36) weighted around 230 ± 15 g were obtained from the National Centre for Laboratory Animal Sciences, ICMR-National Institute of Nutrition, Hyderabad. Animals were housed in individual ventilated cages (IVC) at a temperature (24 ± 2 °C) and humidity (50%) controlled room with a 12 h light/dark cycles and 60–75 air changes per hour. Rats had water and AIN-93 diet as *ad libitum* throughout the study period.

Rats were divided into six groups (*n* = 6). Rats in Group I received the vehicle-phosphate buffer saline (PBS) as control. Group II were administered with fructooligosaccharide (FOS) (5 g/kg-Bw) in PBS which is the standard prebiotic group. Whereas, animals in Groups III, IV, and V were received hydro alcoholic extracts of *O. sanctum, Z. officinale,* and *P. nigrum* in PBS at 850, 500, and 100 mg/kg-Bw, respectively. A combination of three extracts (*O. sanctum* 850 mg/kg-Bw *+ Z. officinale* 500 mg/kg-Bw *+ P. nigrum* 100 mg/kg-Bw) was administered to Group VI rats. All the extracts were given orally for a period of 30 days. Food intake (daily) and body weights (twice-weekly) were monitored throughout the experimental duration.

### Tissue/biosample collection

On day 31 (end of treatment period), ∼1.0 mL of blood (three aliquots) was collected from retro-orbital plexus of each rat, followed by euthanizing of rats for collection of vital organs (liver, kidneys, spleen, heart, lungs, stomach, small intestine and colon) for histopathology analysis. The entire caecum from each rat was collected in sterile container under aseptically conditions, subsequently transferred the caecal content into sterile vials and stored at −20 °C until further process to determine the selective gut bacteria. The blood samples were used for haematology and biochemical analysis.

### Laboratory analysis

#### Haematology

Blood collected in EDTA tubes were used for hematological analysis on automated blood cell counter (Advia 120 automated haematology analyser, Siemens). The haemoglobin (Hb) content, total white blood cells (WBCs), red blood cells (RBCs), differential leukocyte count (DLC), was analysed to assess the effect of extracts on blood parameters.

#### Biochemical assays

The plasma total cholesterol, high density lipoprotein (HDL), low density lipoprotein (LDL), and triglycerides (TG) level were determined by using clinical automated analyser (catalogue number: 06380115, Cobas c 311 analyser ROCHE). Serum lipopolysaccharide (LPS) (catalogue number: CSB-E14247r, Cusabio, Barksdale, DE, USA), Fasting plasma insulin (catalogue number: ELR-Insulin Ray Bio Norcross, GA, USA), serum C-reactive-protein (CRP) and interleukins (IL-6) (catalogue numbers: DY1744(CRP), DY506 (IL-6), R&D Systems, Inc, USA) were quantified using rat ELISA kits as per the manufacturer’s protocols.

### Extraction and purification of DNA from Cecal content

The QIAamp DNA Stool Mini kit (Qiagen 51604) was used to extract DNA from Caecal content of all rats as per manufacturer’s instructions. The quality and quantity of isolated DNA was determined at Å260 nm/Å280 nm using Nanodrop spectrophotometer (Thermo Scientific NanoDrop™ 2000/2000c). DNA Samples were stored at −20 °C until further processing for Real-Time PCR.

### Primers for DNA amplification and quantitative real time polymerase chain reaction (RT-PCR)

The primers used in the investigation are listed in Table 1. RT-PCR reactions were carried out on MicroAmp Fast Optical 96-Well Reaction Plates using a StepOnePlus Real-Time PCR System (Applied Biosystems, CA, and U. S. A). Each reaction contained 2 µL of diluted DNA, 0.5 µL of 10 µM solution of each primer, and 5 µL of Power SYBR Green PCR Master Mix (Applied Biosystems) in a final volume of 10 µL. The PCR amplification reactions were carried out as follows: 6 min at 50 °C, 10 min at 95 °C, followed by 40 cycles of 15 s at 95 °C and 30 s at 55 °C for *Lactobacillus* and 1 min at 60 °C for *Bifidobacterium*. The PCR amplification reactions were carried out as follows: 6 min at 50 °C, 10 min at 95 °C, followed by 40 cycles of 15 s at 95 °C and 1 min at 60 °C for *Bacteroide* and *Firmicutes*. Detection was carried out on a StepOnePlus Real-Time PCR System (Applied Biosystems, CA, USA). To rule out the possibility of primer dimer formation, the amplified product obtained with each primer pair was checked by agarose gel electrophoresis (2% agarose) followed by melting curve analysis of the qRT-PCR amplified products. Each assay was performed in triplicate in the same run. To determine the sensitivity and specificity of the assays, the PCR assays were confirmed using controls. Bacterial estimation was done by qRT-PCR and their relative expression was determined using the ΔΔCt method with all bacterial as an endogenous control gene. The data were presented as the fold change in gene expression normalized to the endogenous reference gene (all bacterial primer) relative to the baseline time-point.

**Table 1.** Primers used in the study for real-time qPCR and size of PCR products.

Target organism	Primer Sequence (5⇒ to 3⇒)	PCR product bp	T°C	Reference
*Lactobacillus group*	**F:** TGGAAACAGRTGCTAATACCC R: GTCCATTGTGGAAGATTCCC	200	55	Frank et al. 2008
*Bifidobacterium*	**F:** GCGTGCTTAACACATGCAAGTC R: CACCCGTTTCCAGGAGCTATT	126	60	Yadav et al. 2013
*Bacteroides*	F: GTCAGTTGTGAAAGTTTGC R: CAATCGGAGTTCTTCGTG	127	60	Ahmed et al. 2007
Firmicutes	**F:** AGYATGTGGTTTAATTCGAAGCA **R:** AGCTGACGACAACCATGCAC	126	60	Yadav et al. 2013
All bacteria	F: ACTCCTACGGGAGGCAGCAGT R: GTATTACCGCGGCTGCTGGCAC	200	60	Ahmed et al. 2007

### Histopathology

Liver, kidneys, lungs, small intestine, spleen, heart, stomach and colon of the sacrificed rats were dissected, removed, washed with normal saline and placed in 10% neutral buffered formalin solution. The fixed specimens were then trimmed, washed and dehydrated in ascending grades of alcohol. The tissue specimens were cleared in xylene and embedded in paraffin by using tissue processor (Citadel 2000, Thermo Scientific). Paraffin embedded blocks were then sectioned at 4–6 microns thickness with help of Leica microtome and then stained with Haematoxylin and Eosin (H and E) by using auto strainer (Tissue-Tek DRS, Sakura). The H and E-stained sections were examined under microscope (Olympus CX41) for morphological changes in the processed tissue, if any.

### Statistical analysis

The mean and standard error of mean (SE) were calculated for all the variables. Descriptive results are presented as Mean ± SE. ANOVA was used to assess significances between the groups of descriptive data by considering the *P* values <0.05 as significant. Dunnett’s test was used to identify the groups that are homogenous with respect to mean. Analysis was performed using windows SPSS software (V22; SPSS Inc, Chicago, IL).

## Results

### Phenolic compounds of *O. sanctum*, *Z. officinale* and *P. nigrum*

**Figure 1.**
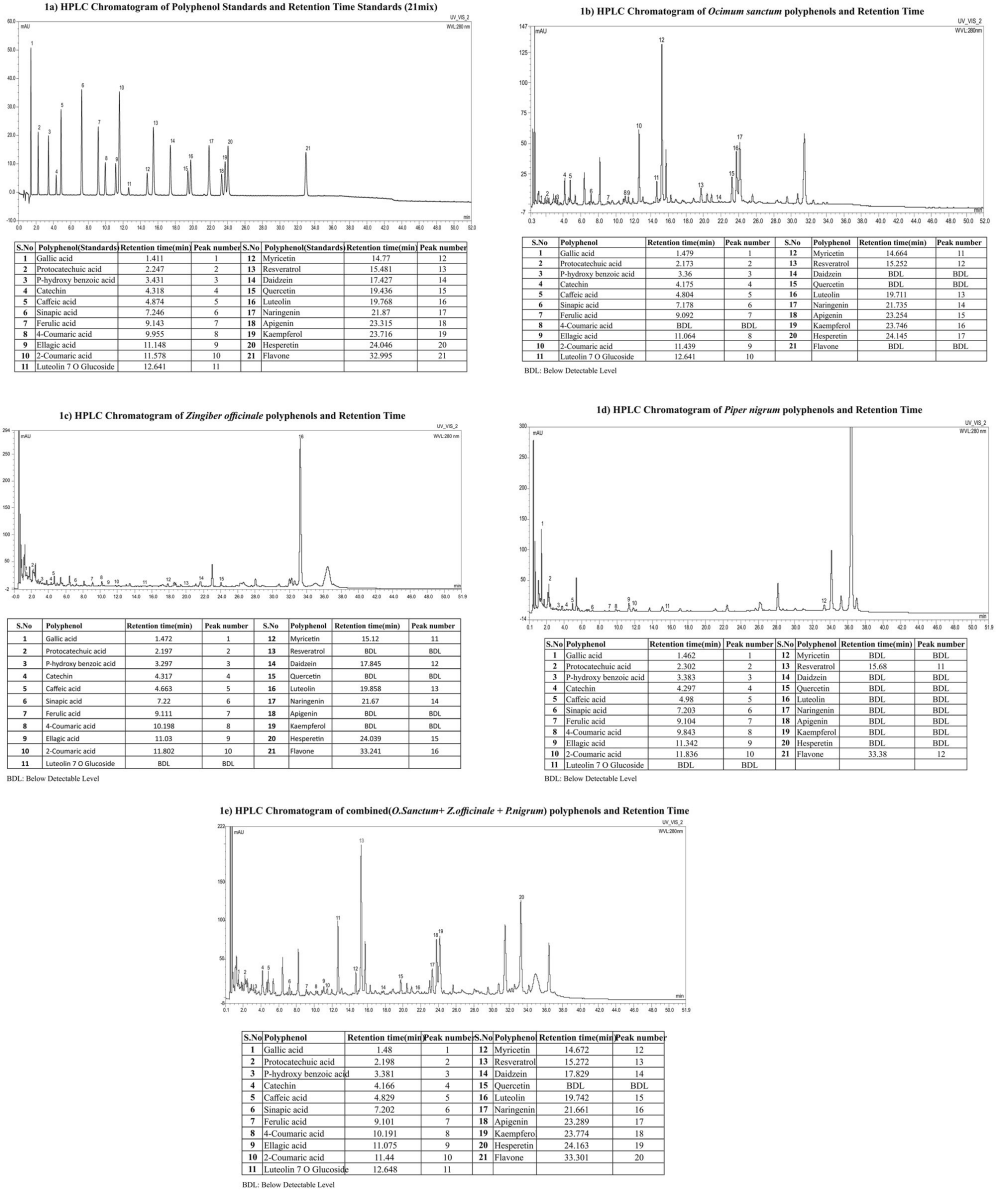
HPLC Chromatograms Polyphenol Standards and Extracts alone and combined and Retention Time %. (a) HPLC Chromatogram of polyphenol Standards and Retention Time. (b) HPLC Chromatogram of *Ocimum sanctum* polyphenols and Retention Time. (c) HPLC Chromatogram of *Zingiber officinale* polyphenols and Retention Time. (d) HPLC Chromatogram of *Piper nigrum* polyphenols and Retention Time. (e) HPLC Chromatogram of combined (*O. Sanctum*+ *Z. officinale* + *P. nigrum*) polyphenols and Retention Time.

The quantitative data of 21 well known individual phenolic compounds and HPLC chromatograms of *O. sanctum, Z. officinale, P. nigrum* and combined extracts were given in Table 2 & Figure 1(a–e). The retention time of individual phenolic compounds were comparable with their respective standards. The luteolin-7-*O*-glucoside (430.0 ± 2.30 mg/100 g), catechin (356.0 ± 2.02 mg/100 g), and resveratrol (270.0 ± 2.10 mg/100 g) contents were higher in *O. sanctum* extract. The *Z. officinale* extract were rich in gallic acid (84.1 ± 2.02 mg/100 g), flavones (21.9 ± 0.91 mg/100 g) and protocatechuic acids (19.3 ± 0.32 mg/100 g) (Table 2). The flavones (88.2 ± 1.80 mg/100 g), catechin (28.2 ± 0.62 mg/100 g) and naringenin (25.0 ± 1.51 mg/100 g) levels were higher in *P. nigrum* extracts. However, in combination of three extracts the luteolin-7-*O*-glucoside (266.0 ± 1.80 mg/100 g), catechin (194.0 ± 1.66 mg/100g) and hesperetin (93.7 ± 2.56 mg/100 g) levels were higher (Table 2; Figure 1(a–e)). The phenolic contents in combination of three extracts are either representing the percentage contributions of each extract or varying; this might be due to interference between one component of extract with other components of another extract or acceptable methodological variations. In addition, the 3 D-HPLC analysis suggests that some ingredients are either increased or disappear on the addition of one extract to other extracts of multi-herbal combinations (Kiyohara et al. 2004).

**Table 2.** Polyphenols in *O.sanctum, Z.officinale, P.nigrum* and combined extracts.

Polyphenols (mg/100gm) (Mean ± SE)	* O. sanctum*	* Z. officinale*	*P. nigrum*	Combination*
Gallic acid	4.02 ± 0.12	84.13 ± 1.2	6.49 ± 0.52	12.91 ± 0.47
Protocatechuic acid	23.53 ± 0.1.2	19.26 ± 0.32	15.38 ± 0.25	22.18 ± 0.62
*p*-hydroxi benzoic acid	12.61 ± 0.32	0.77 ± 0.11	3.35 ± 0.42	8.59 ± 0.43
Catechin	356 ± 2.02	9.70 ± 0.14	28.15 ± 0.62	194 ± 1.66
Caffeic acid	55.68 ± 1.21	1.43 ± 0.01	3.92 ± 0.23	33.32 ± 1.32
Sinapic acid	18.58 ± 0.12	0.95 ± 0.06	2.54 ± 0.31	12.41 ± 0.91
Ferulic acid	7.17 ± 0.23	2.82 ± 0.35	10.45 ± 0.64	8.17 ± 0.33
*p*-Coumaric acid-4	BDL	3.84 ± 0.11	0.80 ± 0.01	0.82 ± 0.04
Ellagic acid	18.14 ± 0.21	14.64 ± 10.62	2.47 ± 0.26	11.39 ± 0.62
*o*-Coumaric acid-2	10.19 ± 0.96	7.06 ± 0.14	1.60 ± 0.02	5.22 ± 0.34
Luteolin-7*O*-Glucoside	430 ± 2.3	BDL	BDL	266 ± 1.8
Myricetin	43.70 ± 1.2	BDL	2.52 ± 0.34	27.52 ± 0.56
Resveratrol	270 ± 2.1	2.72 ± 0.12	BDL	164 ± 2.8
Daidzein	BDL	BDL	2.26 ± 0.41	3.13 ± 0.24
Quercetin	BDL	BDL	BDL	BDL
Luteolin	64.10 ± 1.02	BDL	2.23 ± 0.61	39.09 ± 0.99
Naringenin	6.43 ± 0.31	BDL	24.95 ± 1.51	12.99 ± 0.53
Apigenin	132 ± 3.11	BDL	BDL	79.57 ± 1.65
Kaempferol	57.03 ± 1.21	BDL	BDL	36.78 ± 0.87
Hesperetin	164 ± 2.31	BDL	7.06 ± 0.97	93.66 ± 2.56
Flavone	BDL	21.89 ± 0.91	88.18 ± 1.8	33.36 ± 1.65

*Combination (animal dose: 1450 mg/kg.bwt) = *O. sanctum* (850 mg [58.6%]) + *Z. officinale* (500 mg [34.6%]) + *P. nigrum* (100 mg [7.0%]*;* BDL: Below Detectable Level.

### Chemical composition of essential oils of *O. sanctum*, *Z. officinale* and *P. nigrum*

The percent of essential oil present in *O. sanctum, Z. officinale,* and *P. nigrum* extracts were reported in Figure 2. A vast list of various essential oils noted in *O. sanctum, Z. officinale, P. nigrum* and combination (*O. sanctum + Z. officinale + P. nigrum*) extracts were 38, 151, 56, and 35, respectively (Figure 2). The major essential oil compounds in *O. sanctum* were methyleugenol (13.96%), 1,3,5-cycloheptatriene (11.75%), 2,4-diamino-5-[3,4]-*p*-chlorobenz (9.25%), (*E*)-4-[-(2-benzothiazolyl) etheny (7.11%), eugenol, *N*,*N′*-*bis*(4-chlorobenzylidene)benzene (5.61%), caryophyllene oxide (3.64%) (Figure 2(a)).

Figure 2.Chemical composition of essential oils of *Ocimum sanctum*, *Zingiber officinale* and *Piper nigrum*. (2a) GC-MS Chromatogram of *O. sanctum* essential oils. (b) GC-MS Chromatogram of *Zingiber officinale* essential oils. (c) GC-MS Chromatogram of *P. nigrum* essential oils. (d) GC-MS Chromatogram of *O. sanctum* + *Z. officinale* +*P. nigrum* essential oils.

**Figure:**
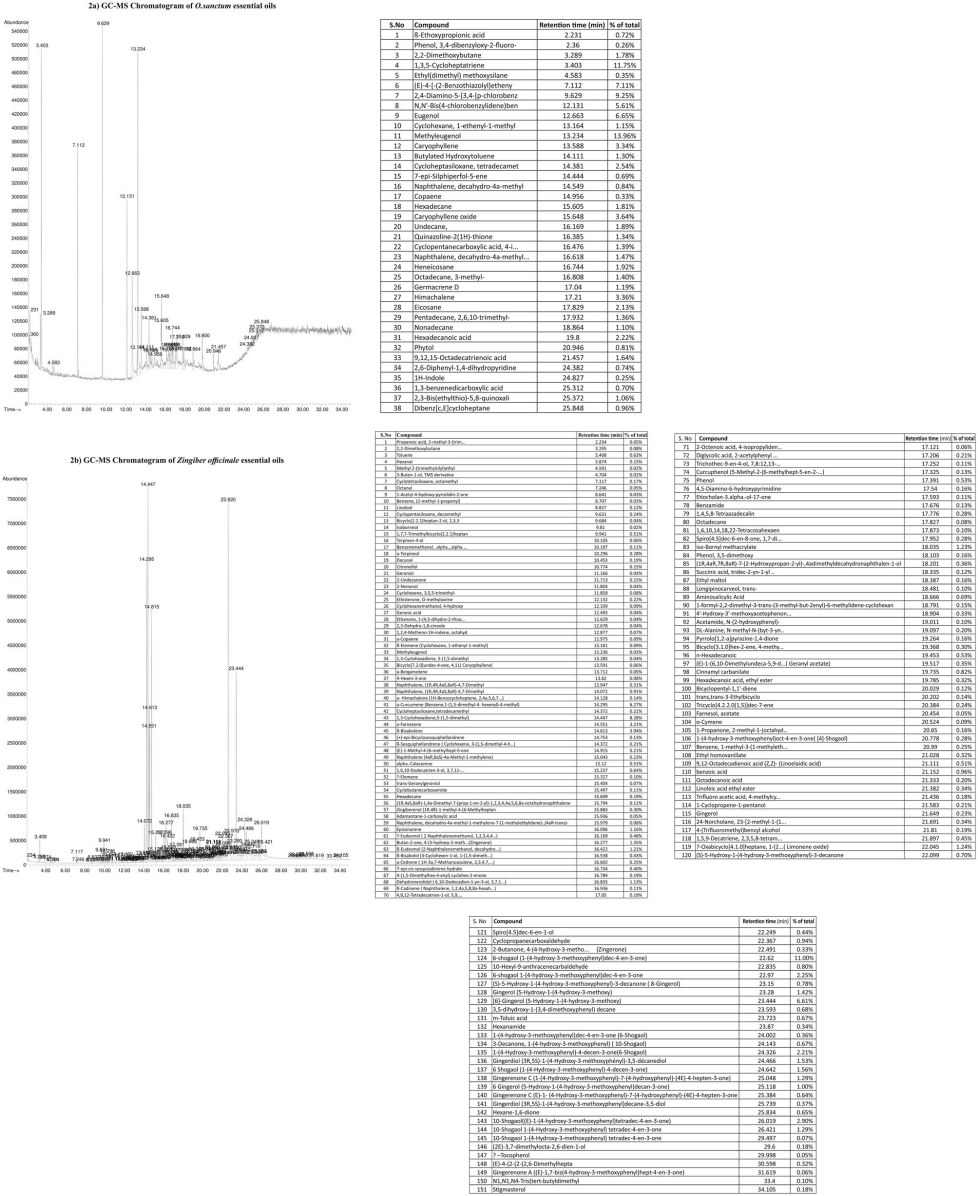

**Figure:**
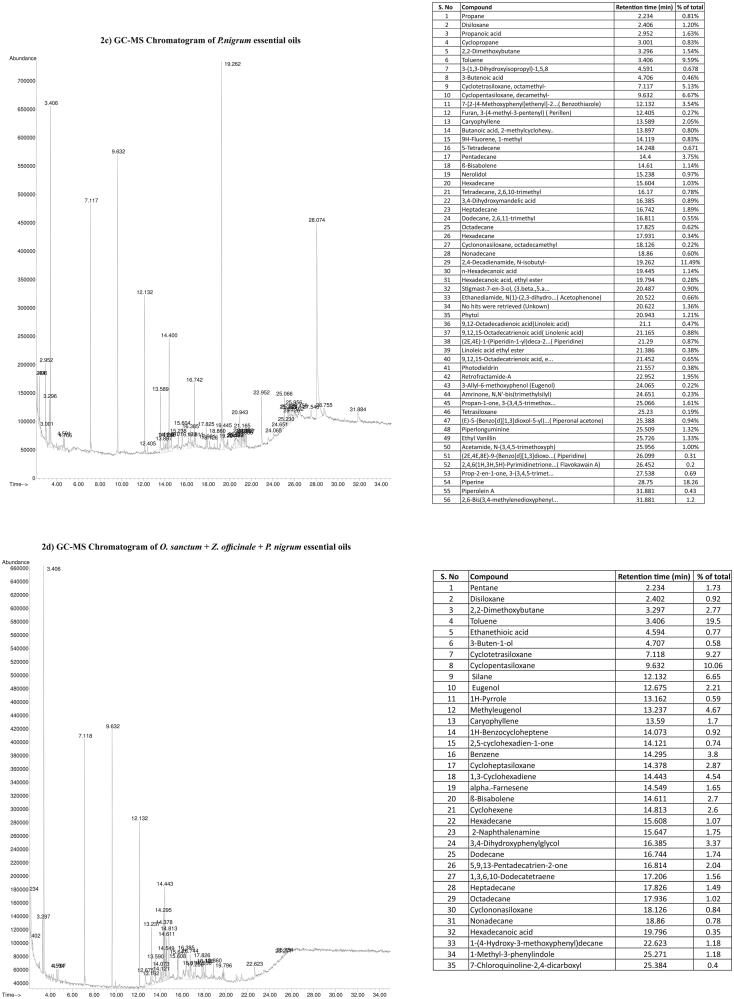

The *Z. officinale* extracts contain 6-shogaol (1-(4-hydroxy-3-methoxyphenyl)dec-4-en-3-one) (11.00%), 1,3-cyclohexadiene,5-(1,5-dimethyl) (8.28%), cyclopentasiloxane, decamethyl (6.61%), α-curcumene (benzene,1-(1,5-dimethyl-4- hexenyl)-4-methyl) (6.266%), β-bisabolene (3.94%), α-farnesene (3.21%), 10-shogaol [(E)-1-(4-hydroxy-3-methoxyphenyl)tetradec-4-en-3-one] (2.90%), 6-shogaol 1-(4-hydroxy-3-methoxyphenyl)dec-4-en-3-one (2.25%), 1-(4-hydroxy-3-methoxyphenyl)-4-decen-3-one(6-shogaol) (2.21%), gingerdiol (3 *R,5S*)-1-(4-hydroxy-3-methoxyphenyl)-3,5-decanediol(1.56%) (Figure 2(b)).

Similarly, the major compounds noted in *P. nigrum* were piperine (18.26%), 2,4-decadienamide, *N*-isobutyl- (11.49%), cyclopentasiloxane, decamethyl- (6.67%), cyclotetrasiloxane, octamethyl-(5.13%), pentadecane (3.75%), 7-[2-(4-methoxyphenyl)ethenyl]-2… (benzothiazole) (3.54%), caryophyllene (2.05%) (Figure 2(c)).

The major compounds in combination (*O. sanctum + Z. officinale + P. nigrum*) extracts were cyclopentasiloxane (10.06%), cyclotetrasiloxane (9.27%), silane (6.65%), methyleugenol (4.67%), 1,3-cyclohexadiene (4.54%), benzene (3.8%), 3,4-dihydroxyphenylglycol (3.37%), 1,3-cyclohexadiene (2.87%), β-bisabolene (2.77%), 2,2-dimethoxybutane (2.7%) (Figure 2(d)).

### Effect of *O. sanctum*, *Z. officinale*, and *P. nigrum* on food intake and body weights

**Figure 3.**
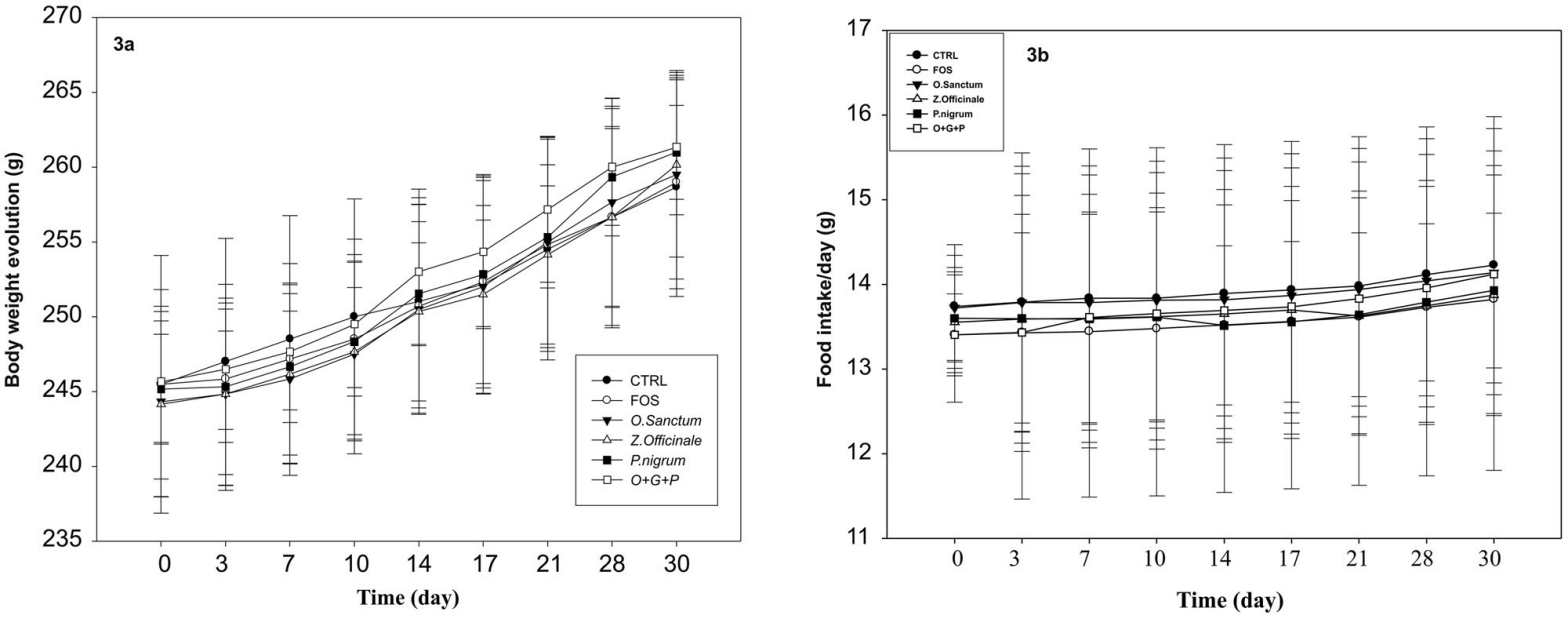
Effect of *O. sanctum, Z. officinale* and *P. nigrum* on bodyweight and food intake.

A proportionate increase in food intake and body weight was noted during the course of experiment irrespective of treatment regimen. The body weights and food intake of rats are given in Figure 3(a, b). The gain in body weight and food intake were comparable among the groups.

### Effect of *O. sanctum*, *Z. officinale*, and *P. nigrum* on haematology parameters

Blood haemoglobin, total white blood cells (WBCs), red blood cells (RBCs), neutrophils, eosinophils, basophils, lymphocyte and monocytes population counts are given in Table 3. A significant (*p* < 0.05) increase in haemoglobin level was noted among *O. sanctum* and combined treated groups compared to the control. Haemoglobin levels of other groups (FOS, *Z. officinale,* and *P. nigrum*) were comparable with the control group (Table 3). The mean levels of total WBCs, RBCs, neutrophils, lymphocytes, monocytes, eosinophils, basophils in FOS, *O. sanctum, Z. officinale,* and *P. nigrum* alone and combined treated rats were compared with controls.

**Table 3.** Effects of *O. sanctum, Z. officinale* and *P. nigrum* on haematology parameters.

	Groups					
Parameters (Mean ± SE)	CTRL	FOS	*O. Sanctum*	*Z. Officinale*	*P. nigrum*	*O. Sanctum + Z. Officinale + P. nigrum*
WBC (10E^3^/mm^3^)	6.10 ± 0.51	6.17 ± 0.49	6.21 ± 0.69	6.11 ± 0.11	6.11 ± 0.22	6.08 ± 0.11
RBC (10E^3^/mm^3^)	8.56 ± 0.14	8.56 ± 0.26	8.51 ± 0.09	8.44 ± 0.22	8.25 ± 0.18	8.48 ± 0.12
Hb (g/dL)	16.75 ± 0.15	17.17 ± 0.40	17.50 ± 0.10*	16.92 ± 0.19	17.02 ± 0.27	17.42 ± 0.12*
Neutrophils (%)	27.50 ± 1.54	28.83 ± 2.01	29.67 ± 0.76	29.33 ± 1.20	29.67 ± 3.11	29.67 ± 2.80
Lymphocytes (%)	65.50 ± 1.89	64.00 ± 1.65	63.50 ± 3.14	62.83 ± 1.35	63.50 ± 3.12	64.83 ± 1.38
Monocytes (%)	0.43 ± 0.04	0.47 ± 0.05	0.48 ± 0.03	0.48 ± 0.03	0.47 ± 0.02	0.48 ± 0.03
Eosinophils (%)	0.27 ± 0.04	0.28 ± 0.05	0.28 ± 0.05	0.28 ± 0.04	0.22 ± 0.02	0.28 ± 0.5
Basophils (%)	0.23 ± 0.03	0.23 ± 0.05	0.23 ± 0.02	0.22 ± 0.02	0.22 ± 0.03	0.22 ± 0.03

Values are Mean ± SE; *The mean difference is significant at the p* < 0.05 (Significant difference between control and groups at 5% level).

### Effect of *O. sanctum*, *Z. officinale*, and *P. nigrum* on lipid profile

**Figure 4.**
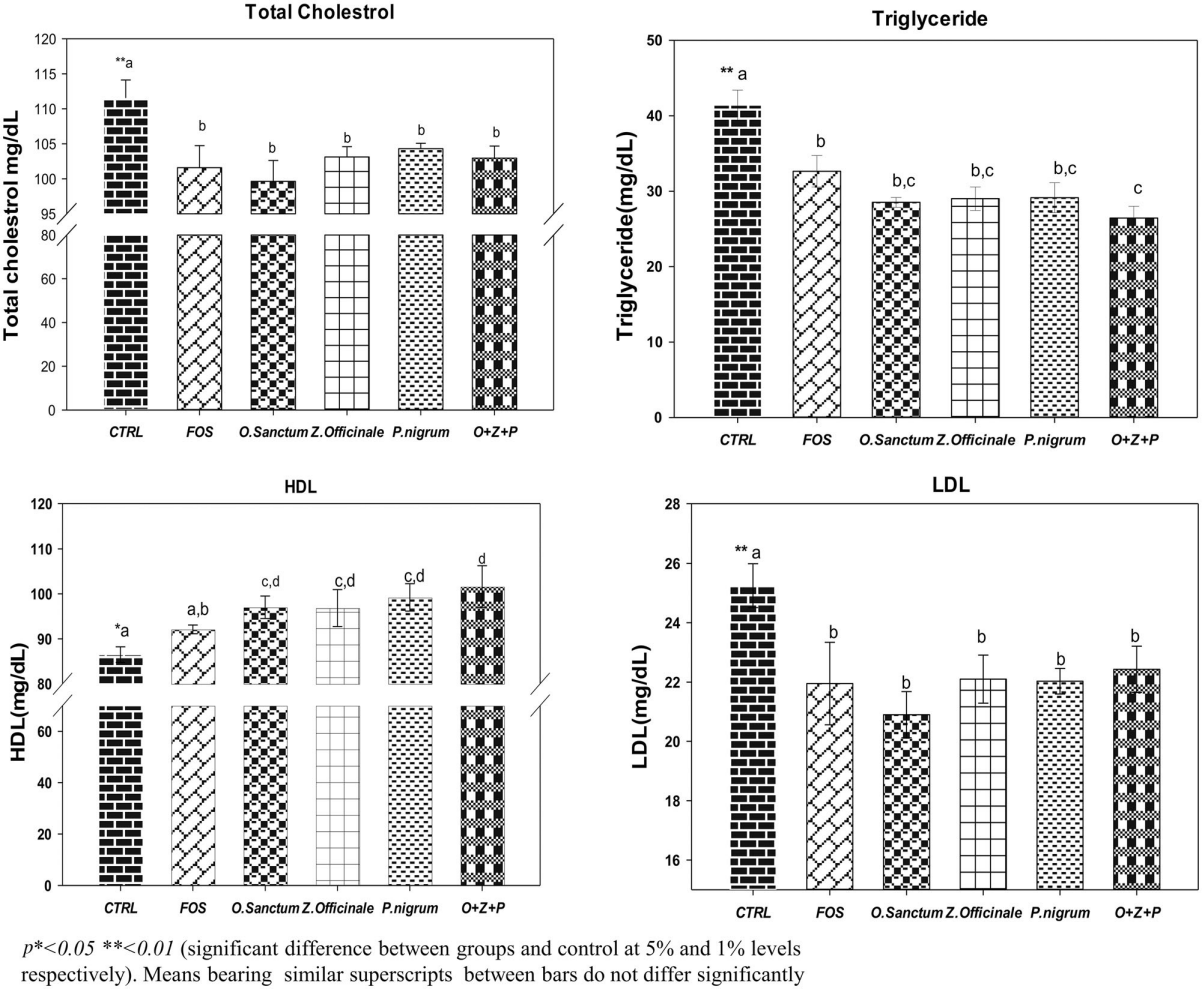
Effect of *O. sanctum, Z. officinale* and *P. nigrum* on lipid profile.

The mean plasma total cholesterol, TG, LDL and HDL levels of rats are given in Figure 4. A significant (*p <* 0.05) decrease in total cholesterol levels were noted in all the groups treated with extracts alone and in combination, as well as the positive control i.e. FOS group, when compared to control (Figure 4(a)). Similarly, the mean TG levels were significantly (*p <* 0.05) lowered in all the rats treated with extracts and FOS administered in comparison to control. Interestingly, TG levels of rats undergone combination treatment were lower (*p <* 0.05) than the FOS treated rats (Figure 4(b)). Similarly, the HDL concentration of rats treated with HA extracts alone and in combination were significantly (*p <* 0.05) higher when compared to both of controls and FOS groups (Figure 4(c)). The mean LDL levels were significantly (*p <* 0.05) low in FOS, extracts administered either alone or in combination when compared to control (Figure 4(d)).

### Effect of *O. sanctum*, *Z. officinale*, and *P. nigrum* on LPS, insulin, CRP & IL-6

**Figure 5.**
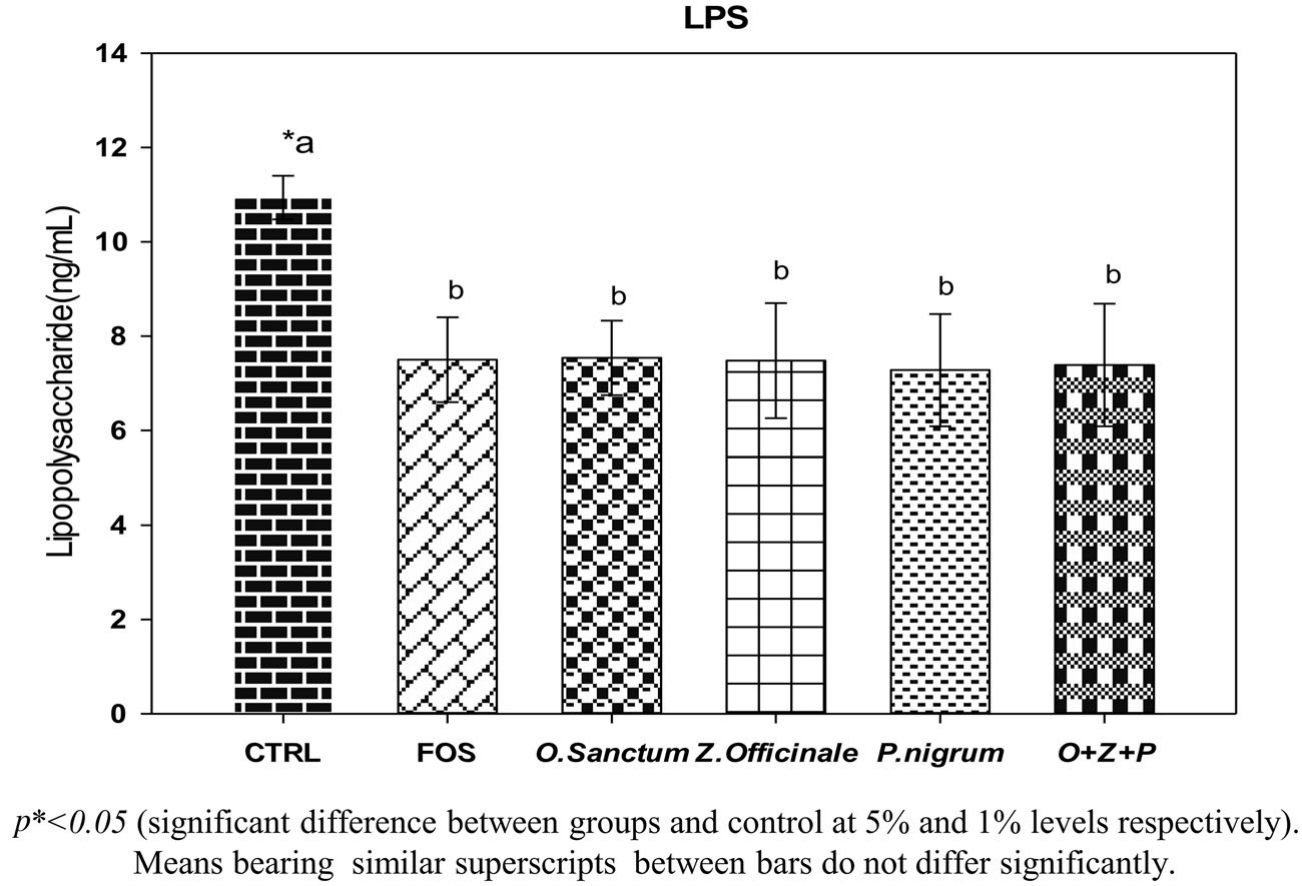
Effect of *O. sanctum, Z. officinale* and *P. nigrum* on LPS.

The mean serum LPS levels of rats are given in Figure 5. A significant (*p <* 0.05) lower levels of serum LPS, a marker of Gram negative bacteria translocation from lumen to circulation, was noted among FOS and extract administered (alone and combined) rats when compared to controls. However, the LPS levels were comparable among the FOS and extract administered rats (Figure 5). Serum insulin, CRP and IL-6 levels are given in Table 4. A minimal increase in mean insulin levels were noted in all the treatments, including FOS compared to control, which is statistically insignificant. Mean CRP levels of serum, a marker of systemic inflammation, were lower in *Z. officinale, P. nigrum* treated rats and FOS, however no significant difference between groups when comparable to controls. Serum IL-6 level decreased in *O. sanctum, Z. officinale, P. nigrum* alone and rats treated with combination and FOS, however, there was no significant difference between groups when compared to control group (Table 4).

**Table 4.** Effects of *O. sanctum, Z. officinale* and *P. nigrum* on CRP, IL-6 & insulin.

Parameters (Mean ± SE)	CTRL	FOS	*O. Sanctum*	*Z. officinale*	*P. nigrum*	*O. Sanctum + Z. officinale + P. nigrum*
C-reactive protein (µg/mL)	2910.16 ± 7.17^a^	2874.64 ± 97.36^a^	2993.94 ± 352.82^a^	2855.78 ± 418.76^a^	2891.72 ± 439.53^a^	2976.15 ± 253.91^a^
IL-6 (pg/mL)	248.7 ± 57.06^a^	228.12 ± 70.41^a^	243.14 ± 61.79^a^	225.52 ± 93.45^a^	227.22 ± 70.71^a^	228.13 ± 79.84^a^
Insulin (IU/mL)	10.13 ± 0.26^a^	13.98 ± 2.03^a^	12.64 ± 0.31^a^	11.51 ± 0.86^a^	14.68 ± 2.12^a^	11.10 ± 1.98^a^

Values are Mean ± SE; Means bearing similar superscripts in each row do not differ significantly.

### Effect of *O. sanctum*, *Z. officinale* and *P. nigrum* on Cecal Lactobacilli, bifidobacterium firmicutes and bacteroides levels

**Figure 6.**
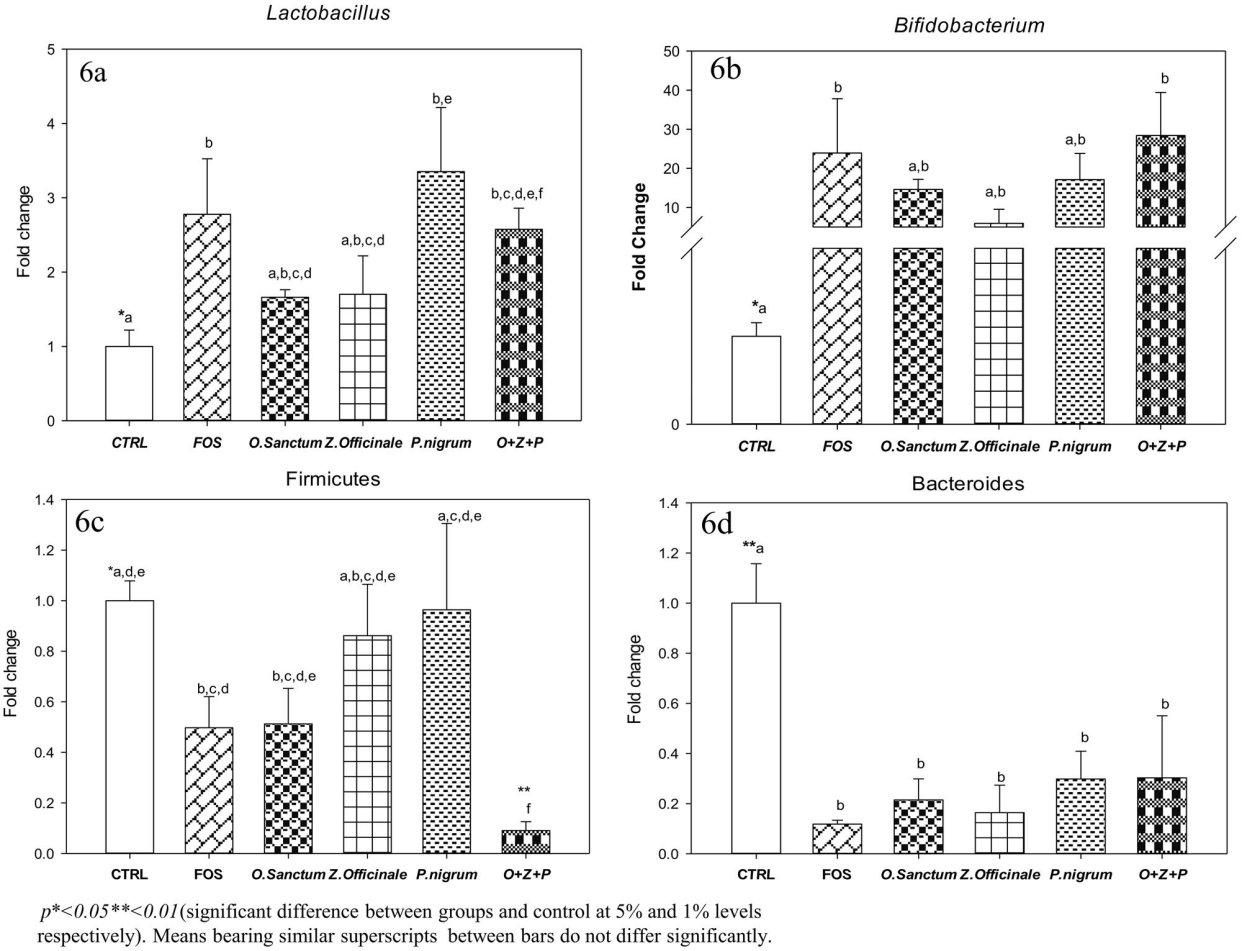
Effect of *O. sanctum, Z. officinale* and *P. nigrum* on Caecal bacterial levels.

The fold change in caecal *Lactobacillus, Bifidobacterium, Firmicutes* and *Bacteroides* are given in Figure 6. Caecal *Lactobacillus* levels increased in rats treated with *O. sanctum, Z. officinale* and *P. nigrum* alone and in combination when compared to control. The fold changes of *Lactobacillus* levels in FOS, *O. sanctum, Z. officinale* and *P. nigrum* treated alone and in combination were 2.7 ± 0.74, 1.66 ± 0.10, 1.7 ± 0.51, 3.35 ± 0.86, and 2.52 ± 0.28-fold, respectively. However, significant (*p <* 0.05) elevation of caecal *Lactobacillus* levels were observed in FOS (∼1.7 fold), *P. nigrum* alone treated (∼2.35 fold) and rats treated in combination (∼1.52 fold) when compared with controls. Whereas, *Lactobacillus* levels in *O. sanctum, Z. officinale* treated rats were comparable to well known prebiotic FOS treated rats and as well as control rats (Figure 6(a)).

Caecal *Bifidobacterium* levels increased in rats treated with *O. sanctum, Z. officinale, P. nigrum* alone and in combination when compared to control rats. *Bifidobacterium* levels in FOS, *O. sanctum, Z. officinale* and *P. nigrum* alone and combined treated rats were 24 ± 13.8, 14.6 ± 2.58, 5.89 ± 3.59, 17.0 ± 6.70 and 28.4 ± 10.9-fold, respectively, when compared to control rats. However, significant (*p <* 0.05) increase in *Bifidobacterium* levels were noted in rats treated in combination (∼27.4-fold) and FOS (∼23-fold) when compared to controls. Though statistically insignificant, an increase in *Bifidobacterium* levels were observed among rats treated with *O. sanctum, Z. officinale,* and *P. nigrum* alone when compared to controls (Figure 6(b)).

Caecal *Firmicutes* levels decreased in FOS, *O. sanctum* alone and combined treated rats when compared to control rats (Figure 6(c)). The fold change of *Firmicutes* levels in FOS; *O. sanctum, Z. officinale,* and *P. nigrum* alone and combined treated rats were 0.51 ± 0.12, 0.49 ± 0.14, 0.14 ± 0.20, 0.04 ± 0.34 and 0.91 ± 0.03-fold, respectively, in comparison to controls. However, *Firmicutes* levels were decreased significantly (*p <* 0.05) in FOS (∼−0.49-fold), *O. sanctum* (∼−0.51-fold) alone and combined treated rats (*p <* 0.01and ∼−0.09-fold) when compared to control rats. Interestingly, the *Firmicutes* levels of combined treated rats were significantly (*p <* 0.01) decreased in comparison to the well-known prebiotic FOS group (Figure 6(c)).

The caecal *Bacteroides* levels were decreased in all the treatment groups including FOS when compared to control rats. The fold decrease in *Bacteroides* levels of FOS, *O. sanctum, Z. officinale,* and *P. nigrum* alone and combined treated rats were 0.88 ± 0.01, 0.78 ± 0.08, 0.83 ± 0.10, 0.70 ± 0.11 and 0.69 ± 0.24-folds, respectively, with reference to controls. A significant (*p <* 0.05) decrease in *Bacteroides* levels were noted among FOS, *O. sanctum, Z. officinale,* and *P. nigrum* alone and combined treated rats in comparison to control rats. It is noteworthy to mention that the *Bacteroides* levels in *O. sanctum, Z. officinale, P. nigrum* and combined treated rats comparable to the well-known prebiotic FOS group (Figure 6(d)).

### Effect of *O. sanctum*, *Z. officinale*, and *P. nigrum* on histopathology

**Figure 7.**
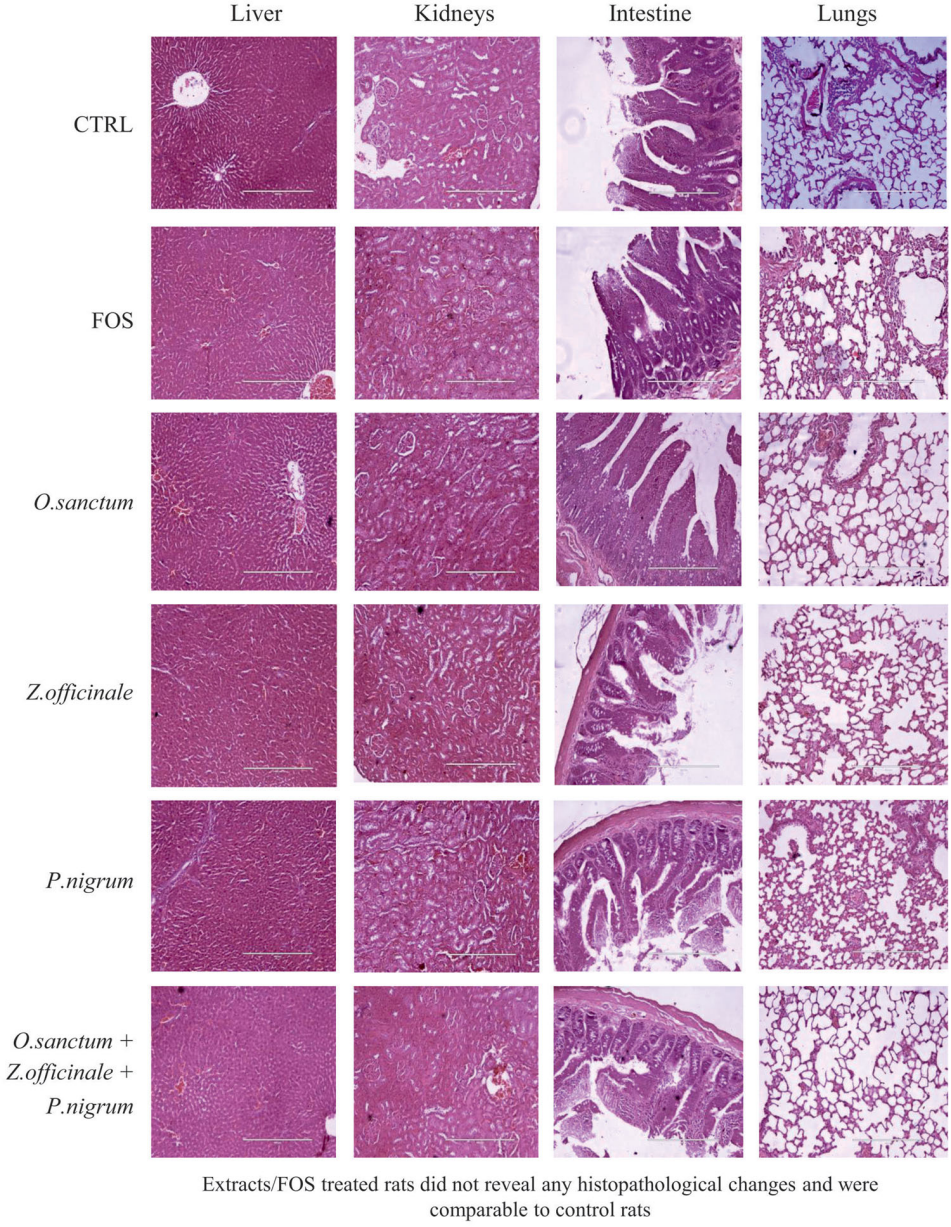
Effect of *O. sanctum*, *Z. officinale* and *P. nigrum* on histopathological observation of vital organs.

Histopathological examination of liver, kidneys, small intestine, lungs, spleen, heart, stomach, and colon of the FOS, *O. sanctum, Z. officinale,* and *P. nigrum* alone and combined treated rats did not reveal any pathological changes and were compared to control group (Figure 7; liver, kidneys, small intestine and lungs only).

## Discussion

To the best of our knowledge, this is the first study reporting the *in vivo* treatments of *O. sanctum, Z. officinale,* and *P. nigrum* hydro-alcohol extract and its effects on healthy rodent’s gut microbiota. Our previous *in vitro* study showed enhanced growth of beneficial microbes, *Lactobacillus* and *Bifidobacterium* in presence of hydro-alcoholic extracts of *O. sanctum, Z. officinale, and P. nigrum* (Babu et al. 2018). RT-PCR based enumeration of gut microbiota of present study involves the *Firmicutes* at phylum level, along with popular genus of the same phylum i.e., *Lactobacillus*; in addition to genus of other phylum includes *Bacteroides* and *Bifidobacterium*. In the current study, the extracts showed remarkable higher levels of *Lactobacillus* and *Bifidobacterium*, whereas an inhibitory effect on *Bacteroides* and *Firmicutes* indicating the prebiotic effect over the conventional prebiotic, FOS, which have been commonly used for many conditions. It is established fact that the FOS is a popular prebiotic, stimulates the growth of beneficial bacteria and is known to improve blood sugar, serum cholesterol, insulin sensitivity and amelioration of inflammation (Markowiak and Śliżewska 2017). A consistent observation of increased *Lactobacillu*s levels with decreased levels of *Firmicutes* among the extract-administered groups (alone/combination), could be attributed to positive effects of phytoconstituents and a complex association between different genera at GI tract. The phylum *Firmicutes* are comprised of ∼95% with *Clostridium genera*, whereas the remaining 5% are *Lactobacillus, Bacillus, Enterococcus,* and *Ruminicoccus* (Rinninella et al. 2019). Further, according to ‘enterotype’ classification, the *Lactobacillus* is the co-occurring members of the ‘enterotype-1′ (Bacteriodes) which has an inverse association with genus *Bacteroides* and *Clostridium* (Arumugam et al. 2011). Results of current study corroborated with these observations wherein the *Lactobacillus* levels are increasing with extracts with overall decrease in *Firmicutes*, owing to that the genus *Clostridium* is main contributor.

The higher prebiotic potential of *O. sanctum, Z. officinale* and *P. nigrum* as observed in the current study suggests greater role for these herbs in health promotion. The prebiotic potential of the herbs could be attributed to the presence of significantly higher concentrations of phytochemicals/polyphenols/essential oils (Duenas et al. 2015; Liu et al. 2017; Babu et al. 2018). In addition to polyphenols/essential oils, the oligosaccharides present in the extracts may contribute to the growth of beneficial bacteria (*Lactobacillus* and *Bifidobacterium*) and inhibition of *Bacteroides* (Markowiak and Śliżewska 2017). Essential oils are natural bioactive compounds derived from plant and have positive effects on growth and health (Puvača et al. 2013). The importance of bioactive components of essential oils has been gaining momentum and advancement in technology results in understanding the mechanism of action. A few of the studies conducted on normal healthy chicks and swine by supplementation of essential oils confirms the antioxidant and anti-inflammatory properties of essential oils, along with improvement in gut microbiome (Liu et al. 2017; Omonijo et al. 2018). Quantitative analysis of essential oil composition of selected extracts of present study reveals that presence of a wide range (∼38 to ∼151) of bioactive components in the herbs. The positive effect of extracts administration on gut microbiome in terms of significantly higher levels of *Lactobacillus* and *Bifidobacterium* along with reduced levels of *Firmicutes* and *Bacteroides* could be due to a wide variety of bioactive components of essential oils.

A constant observation of improvement in haemoglobin levels was noted with *O. sanctum* administration, which is in line with our earlier study (Hemalatha et al. 2011). Similar to vast published reports, results of current study showed that oral administrations of individual extracts of *O. sanctum, Z. officinale* and *P. nigrum* and even the additive dose of three extracts i.e., combined treatment (1450 mg/kg-Bw: *O. sanctum* 850 mg/kg-Bw *+ Z. officinale* 500 mg/kg-Bw *+ P. nigrum* 100 mg/kg-Bw) are safe and have no adverse effects on vital organs and hematological profile (Chunlaratthanaphorn et al. 2007; Rong et al. 2009; Gautam and Goel 2014; Sriwiriyajan et al. 2016; Singh and Chaudhuri 2018). Best of our knowledge this might be the first study of its kind in testing the additive effect of these selected herbs on healthy rats.

Prebiotic effects have been observed in animal model studies with tea polyphenols, red wine, polyphenols powder, apple juice, resveratrol, blackcurrant extracts with abundant growth of *Lactobacillus, Bifidobacterium* and decreased growth of *Bacteroides* (Duenas et al. 2015). In addition to the prebiotic potential of selected herbal extracts, current study has shown a favourable regulation of lipid profile. This finding was corroborated with a study conducted in normal albino rabbits fed on diet mixed with fresh leaves of *O. sanctum* (1–2 g/100 g of diet/4 weeks) resulted in significant lowering of plasma phospholipid, TG, total and LDL-cholesterol levels, with significant increase in HDL-cholesterol (Sarkar et al. 1994). Interestingly many studies have shown favourable regulation of lipid profile along with anti-diabetic and anti-hyperlipidemic activities with these three selected herbal extracts (Alizadeh-Navaei et al. 2008; Damanhouri and Ahmad 2014; Parasuraman et al. 2015; Sarfaraz et al. 2016). These findings clearly suggest that herbal extracts of present study have immense prebiotic potential, apart from regulating the intestinal bacteria, thereby improving the health status.

Plant foods possess cholesterol-suppressive capacity (Alizadeh-Navaei et al. 2008). Previous reports have shown that the plant food-derived ingredients, β-carotene and lycopene, also act as hypocholesterolemic agents, secondary to their inhibitory effect on cellular cholesterol biosynthesis (Alizadeh-Navaei et al. 2008). Several lines of evidence showed that plants with phenolic compounds had antilipidemic and antioxidant activities, which may aid in protecting liver and heart (Alizadeh-Navaei et al. 2008).

Although statistically not significant, there was an improvement in insulin levels with *O. sanctum, Z. officinale,* and *P.nigrum*. These findings are in line with observations reported by other researchers (Kaleem et al. 2005; El-Kott et al. 2010; Hannan et al. 2015). *O. sanctum, Z. officinale,* and *P. nigrum* have been shown to bring down the CRP levels in various disease conditions in both experimental animals and humans (Ahmed et al. 2013; Kavitha et al. 2015; Mazidi et al. 2016). Anti-inflammatory potential of these herbs has been demonstrated both in humans and cellular models of gut inflammation (Damanhouri and Ahmad 2014; Kim et al. 2017; Singh and Chaudhuri 2018). However, in the current study the CRP and IL-6 levels were comparable among all the groups irrespective of treatment regimen. Interestingly, the LPS levels, a marker of Gram negative bacterial load, were significantly decreased upon administration of extracts. As the present study was conducted in normal healthy rats, obliviously there were no changes in the inflammatory markers unlike the above studies wherein the effects of the herbs were studied in inflammatory conditions. An interesting finding of present study is decreased levels of LPS, *Bacteroides* and *Firmicutes,* along with higher levels of *Lactobacillus* and *Bifidobacterium* in extracts administered animals and *vice versa* in control rats suggesting the extracts are possessing the prebiotic activity. Further, this observation is a complex phenomenon existing between the gut microbiome and basal/chronic low-grade inflammation. It is well established fact that chronic low grade inflammation is the underling pathogenesis of non-communicable diseases hence the anti-inflammatory properties of these herbal extracts have been exploring in diseases such as obesity, diabetes, cardiovascular disease and asthma (Laveti et al. 2013). The prebiotic potential, lipid lowering effects, reduction in LPS levels and increase in caecal beneficial bacteria by the herbal extracts of current study could be attributed to the presence of higher concentrations of phytochemicals (polyphenols and essential oils). It is noteworthy to mention here that the present study could be first of its kind in exploring the prebiotoic potential of widely using herbal/spice extracts and their modulatory effects towards the native gut flora in SD rats. Another important aspect of the current study is supplementation of extracts as alone and in combination, wherein the synergistic effect of active phytoconstituents could be responsible for various properties assessed in the study. Nonetheless, the limitation of the present study would be a lack of data with respect to ‘anti-nutrients’, if any, so that translational research would help in developing prebiotic product with a holistic approach.

## Conclusions

The study found that oral administration of these extracts to healthy animals confirms the lipid lowering effects, favourable modulation of selected gut microbiota there by reducing the basal/low grade LPS levels. In conclusion, the extracts are exerting the prebiotic potential, which may be explored in developing of prebiotic products for improvement of gut bacterial alterations.
